# *Arabidopsis* NLR Immune Receptors: Structural Diversification, Signaling Networks, and Emerging Principles of Immune Activation

**DOI:** 10.3390/plants15193007

**Published:** 2026-09-30

**Authors:** Jianzhong Huang

**Affiliations:** 1 Department of Basic Medicine, Fuzhou Medical College, Fuzhou 344000, China; huangjz@whu.edu.cn; 2 College of Life Sciences, Wuhan University, Wuhan 430072, China

**Keywords:** *Arabidopsis* NLR, resistosome, TIR signaling, helper NLR, EDS1, immune receptor engineering, immune signaling network

## Abstract

Plant intracellular nucleotide-binding leucine-rich repeat receptors (NLRs) are central to effector-triggered immunity (ETI), but the rapid expansion of structural data has outpaced an integrated explanation of how receptor architecture, oligomerization, and network context determine immune output. Recent structural and biochemical studies, particularly in *Arabidopsis*, have revealed that NLRs operate as dynamic signaling assemblies rather than simple pathogen-recognition switches. Here, we synthesize recent evidence across coiled-coil NLRs (CNLs), Toll/interleukin-1 receptor NLRs (TNLs), and RPW8-like helper NLRs (RNLs), with emphasis on mechanisms that are broadly conserved and those that remain receptor- or pathway-specific. ZAR1 (HopZ-Activated Resistance1) and selected helper RNLs form membrane-associated Ca^2+^-permeable channels, whereas TNLs use Toll/interleukin-1 receptor (TIR)-domain enzymatic activity to generate signaling molecules that are decoded through branch-specific modules formed by ENHANCED DISEASE SUSCEPTIBILITY1 (EDS1)–PHYTOALEXIN DEFICIENT4 (PAD4)–ACTIVATED DISEASE RESISTANCE1 (ADR1) or EDS1–SENESCENCE-ASSOCIATED GENE101 (SAG101)–N REQUIREMENT GENE1 (NRG1). Paired NLRs partition recognition and signaling, while recent work on ADR1-L1 indicates that higher-order spatial organization can further tune immune execution. We also distinguish structurally resolved complexes from inferred higher-order assemblies, examine crosstalk with cell-surface immunity and transcriptional regulation, and assess NLR-engineering strategies according to their experimental validation and potential fitness costs. Together, the evidence supports a modular view in which oligomerization is a recurrent activation principle, but biochemical output, partner dependence, and membrane execution are not interchangeable across NLR classes.

## 1. Introduction

Plants are continuously exposed to diverse pathogens, including bacteria, fungi, oomycetes, viruses, and nematodes. Because plants lack mobile immune cells, individual cells must rely on intrinsic surveillance systems to detect pathogen-derived molecules and pathogen-induced alterations in host physiology. Plant immunity is traditionally divided into pattern-triggered immunity (PTI), which is initiated by cell-surface pattern-recognition receptors (PRRs), and effector-triggered immunity (ETI), which is mediated primarily by intracellular nucleotide-binding leucine-rich repeat receptors (NLRs) [1,2]. This classification has provided a useful framework for understanding immune activation; however, studies on *Arabidopsis* have revealed extensive interactions between these two layers of defense. Signals initiated by PRRs can influence NLR-mediated immunity, whereas activated NLR pathways can reinforce components associated with surface receptor signaling [3,4]. Therefore, understanding NLR function requires consideration of not only pathogen recognition but also the signaling processes that connect receptor activation with immune execution.

Most *Arabidopsis* NLRs share a conserved modular organization consisting of an N-terminal signaling domain, a central nucleotide-binding NB-ARC domain, and C-terminal leucine-rich repeats (LRRs) [5]. The NB-ARC domain controls nucleotide-dependent conformational transitions, whereas the LRR region contributes to effector recognition, autoinhibition, and receptor assembly [5]. Based on their N-terminal domains, *Arabidopsis* NLRs are broadly classified into Toll/interleukin-1 receptor NLRs (TNLs), coiled-coil NLRs (CNLs), and RPW8-like NLRs (RNLs) [6,7,8]. These receptor classes differ substantially in their mechanisms of immune activation: TNLs rely on TIR-domain-mediated signaling, whereas CNLs and RNLs participate in resistosome formation and downstream immune execution [6,8]. Although the overall NLR architecture is conserved, diversification of individual domains has generated receptors with distinct recognition properties and signaling capacities. One important mechanism underlying this diversification is the acquisition of integrated domains (IDs) [9], which can function as pathogen recognition modules by mimicking host targets manipulated by pathogen effectors. In *Arabidopsis*, the RRS1/RPS4 pair represents a well-characterized example in which an integrated WRKY domain contributes to effector perception and activates immune signaling through its paired receptor partner [10].

Recent structural studies have substantially expanded our understanding of how NLRs execute immune responses. Rather than acting as simple binary switches, activated NLRs undergo extensive conformational remodeling and oligomerization to generate higher-order signaling assemblies termed resistosomes [11,12]. Studies of *Arabidopsis* NLRs have demonstrated that these assemblies can adopt distinct architectures with specialized functions [12,13,14]. CNL resistosomes and activated helper NLR oligomers can form membrane-associated Ca^2+^-permeable channels [12,14], whereas TNLs activate downstream immunity through TIR-domain enzymatic activities that generate nucleotide-derived immune signaling molecules [13,15,16,17,18]. These discoveries indicate that receptor activation, oligomer assembly, and downstream signaling are closely interconnected processes. In addition to individual receptor activation, recent studies have revealed that *Arabidopsis* NLR immunity relies on coordinated interactions among multiple receptor components. Paired NLRs can partition pathogen recognition and signaling execution between functionally specialized receptor partners [19,20], whereas helper NLRs function as shared downstream signaling components for diverse sensor NLRs [14,21,22]. Furthermore, the higher-order clustering of ADR1-L1 resistosomes suggests that spatial organization represents an additional layer of regulation in NLR immune signaling [23].

Several recent reviews have summarized NLR structure, activation, and engineering [6,24,25,26,27]; however, important mechanistic distinctions can be obscured when evidence from different receptor classes and species is discussed together. In particular, channel formation has been demonstrated for specific CNL and RNL systems rather than for every member of these classes, the two EDS1-dependent TNL branches are not biochemically equivalent, and some higher-order assemblies are supported by in planta imaging or biochemical evidence without atomic structures. In this review, we therefore compare conserved and class-specific activation principles across *Arabidopsis* TNLs, CNLs, and RNLs; distinguish resolved structures from working models; and examine how paired receptors, helper-NLR networks, PTI crosstalk, transcriptional regulation, and signaling interference shape immune output. We further assess engineering strategies by separating experimentally validated approaches from prospective designs and by considering partner compatibility, autoimmunity, activation thresholds, and fitness costs.

## 2. Structural Diversification of *Arabidopsis* NLR Immune Receptors

**Figure 1:**
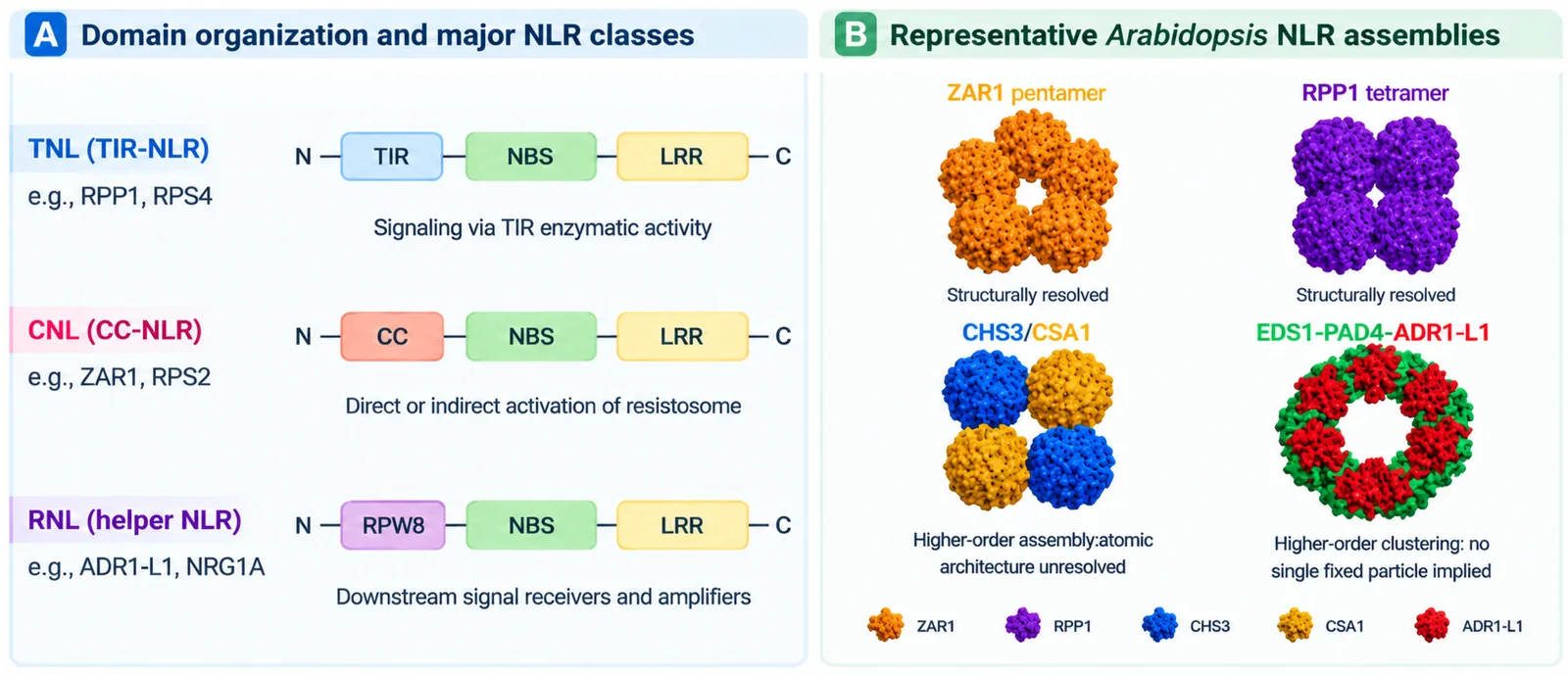
Classification and structural diversity of *Arabidopsis* NLR immune receptors and representative activated assemblies. (**A**) Domain organization and classification of major *Arabidopsis* NLR immune receptors. *Arabidopsis* NLRs are classified into Toll/interleukin-1 receptor NLRs (TNLs), coiled-coil NLRs (CNLs), and RPW8-like NLRs (RNLs) according to their N-terminal signaling domains. All NLR classes share a conserved central nucleotide-binding NB-ARC domain and C-terminal leucine-rich repeat (LRR) region but possess distinct N-terminal modules that determine their signaling properties. TNLs contain TIR domains with enzymatic signaling activity, whereas selected CNLs and RNLs form membrane-associated resistosomes. (**B**) Representative *Arabidopsis* NLR assemblies with evidence status indicated. ZAR1 and RPP1 are shown as experimentally resolved receptor assemblies. CHS3/CSA1 is shown schematically because the atomic architecture of its activated higher-order assembly remains unresolved, whereas the EDS1–PAD4–ADR1-L1 panel represents higher-order clustering observed during signaling rather than a single particle with a defined stoichiometry.

Although *Arabidopsis* NLRs share a conserved receptor architecture, variations within individual domains have generated substantial functional diversity. Most NLRs contain an N-terminal signaling domain, a central nucleotide-binding NB-ARC domain, and C-terminal leucine-rich repeats (LRRs). The NB-ARC domain functions as a molecular switch that regulates nucleotide-dependent conformational transitions, whereas the LRR region contributes to receptor autoinhibition, effector perception, and assembly of activated complexes [5,6,28]. The N-terminal domain is a major structural determinant distinguishing NLR classes and is closely linked to their signaling functions. Accordingly, *Arabidopsis* NLRs are broadly classified into TIR-domain NLRs (TNLs), coiled-coil NLRs (CNLs), and RPW8-like coiled-coil NLRs (RNLs) [6,24] (Figure 1A). TNLs contain N-terminal TIR domains whose enzymatic activities generate immune signaling molecules [15,16,17]. CNLs contain coiled-coil domains and can assemble into resistosomes following activation [12], whereas RNLs, including members of the ADR1 and NRG1 families, function as helper NLRs that mediate downstream signaling from sensor immune receptors [14,22,29]. Thus, conserved domain organization provides a stable structural framework, while diversification of individual domains enables different modes of immune activation.

One important mechanism contributing to NLR diversification is the incorporation of integrated domains (IDs) [30]. These additional domains are often derived from, or homologous to, host proteins targeted by pathogen effectors and can serve as integrated recognition modules or decoys [10,30]. By incorporating pathogen-targeted host structures into immune receptors, NLRs can acquire new recognition capabilities while retaining conserved signaling modules. The RRS1/RPS4 pair provides a well-characterized example of this strategy. RRS1 contains an integrated WRKY domain that mimics host regulatory targets manipulated by pathogen effectors [10,31]. Effector interaction with or modification of this domain activates RPS4-dependent immune signaling, illustrating how structural innovation within an NLR can convert pathogen virulence strategies into immune recognition events [10,19,31].

Structural diversification is also reflected in the distribution of recognition and signaling functions among different NLR components. Some NLRs integrate pathogen perception and immune activation within a single receptor, whereas others depend on receptor partnerships for effective signaling. Paired NLR systems partition these functions between a recognition-specialized sensor and a functionally specialized signaling partner [19], whereas helper-NLR networks further extend this modular organization by enabling multiple sensor receptors to utilize shared downstream signaling components [22,29]. Such modularity may facilitate the diversification of pathogen recognition while preserving conserved immune signaling outputs. At the same time, this functional specialization requires precise coordination among interacting NLR components to ensure productive immune activation.

## 3. Resistosome Assembly Defines Distinct Modes of *Arabidopsis* NLR Activation

In their resting state, many NLRs are maintained in autoinhibited conformations through coordinated interactions among their N-terminal signaling domains, NB-ARC domains, and LRR regions. Direct effector recognition or perception of effector-induced modifications of host proteins relieves these inhibitory constraints, promoting conformational rearrangement, nucleotide-dependent activation, and receptor oligomerization [11,12,13,28]. Here, we use the term resistosome for an activated NLR oligomer whose assembly has been structurally or biochemically supported; when stoichiometry or atomic organization remains unresolved, we refer instead to a higher-order NLR assembly. Across NLR classes, activation commonly involves release of autoinhibition and conformational reorganization, but the biochemical output of the resulting assembly differs markedly among receptor types.

**Figure 2:**
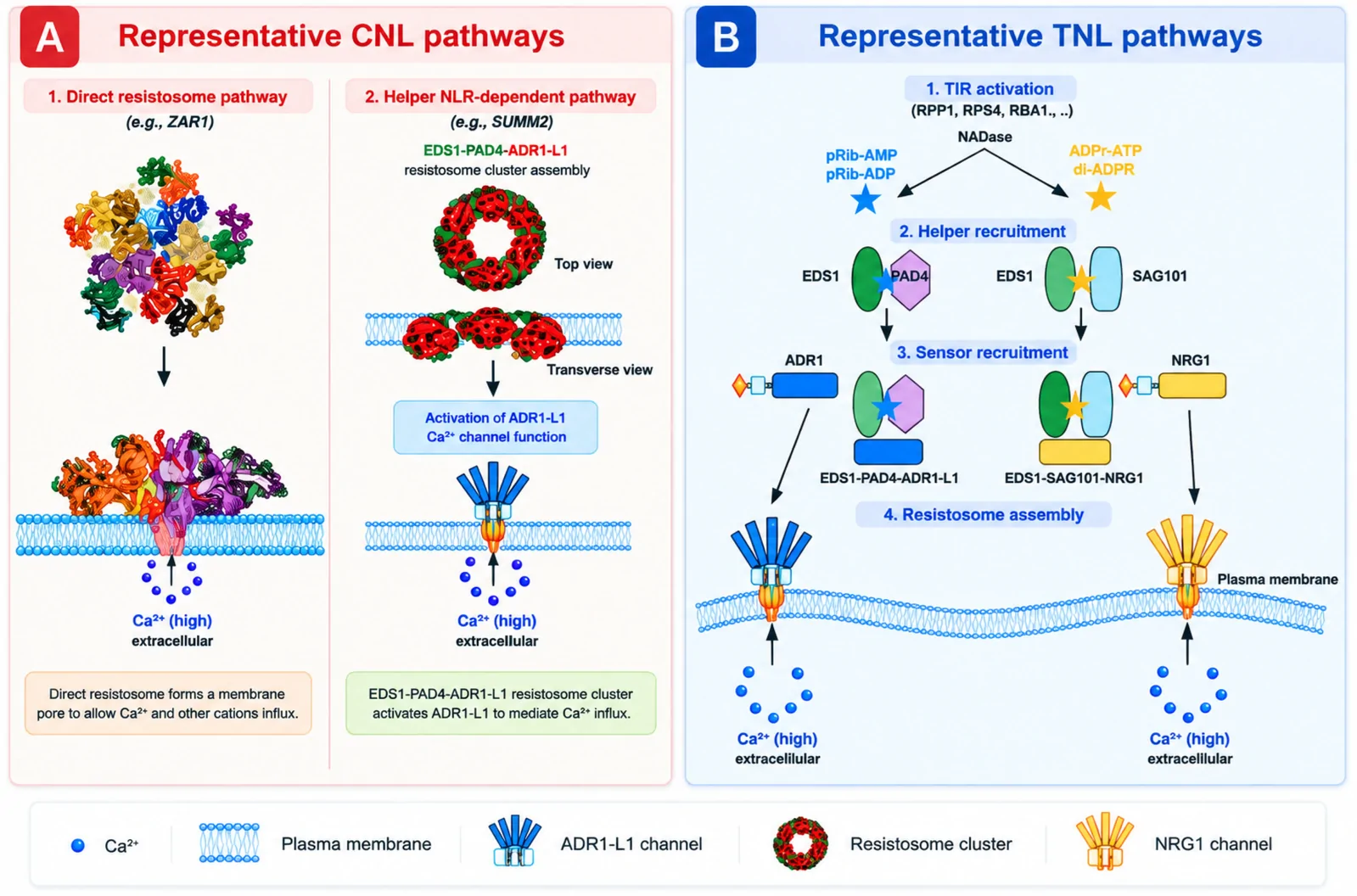
Representative CNL- and TNL-mediated immune signaling pathways in *Arabidopsis*. (**A**) Representative CNL pathways. ZAR1 illustrates direct activation of a membrane-associated resistosome with Ca^2+^-permeable cation-channel activity. In the SUMM2 pathway, the sensor CNL acts upstream of an EDS1–PAD4–ADR1-L1 module and higher-order ADR1-L1 clustering at the plasma membrane. (**B**) Representative TNL pathways. TIR enzymatic activity generates branch-selective signaling molecules: pRib-AMP/ADP activates the EDS1–PAD4–ADR1 branch, whereas ADPr-ATP/di-ADPR activates the EDS1–SAG101–NRG1 branch. Activated helper RNLs can then form plasma-membrane Ca^2+^-permeable channels. These schemes summarize experimentally supported examples and are not intended to imply that every CNL or TNL uses an identical execution mechanism.

Among *Arabidopsis* CNLs, ZAR1 remains the best-characterized example of resistosome-mediated immune activation (Figure 1B). Upon activation, ZAR1 assembles into a pentameric resistosome in which the N-terminal coiled-coil domains undergo extensive rearrangement, exposing α-helical elements that form a membrane-associated pore structure. Electrophysiological and cellular studies have demonstrated that the ZAR1 resistosome functions as a Ca^2+^-permeable cation channel and drives downstream responses including cytosolic Ca^2+^ elevation, reactive oxygen species accumulation, and immune-associated cell death [11,12] (Figure 2A). These data establish membrane pore formation as an experimentally validated execution mechanism for ZAR1. They do not, however, justify treating Ca^2+^-permeable channel formation as a universal property of all *Arabidopsis* CNLs; for many CNLs, oligomeric state and terminal biochemical output remain unresolved.

Not all *Arabidopsis* CNLs execute immune signaling through self-assembled pore-forming resistosomes. The SUMM2 pathway illustrates an alternative mode of CNL-mediated immune activation in which an upstream sensor CNL promotes the assembly of downstream helper-NLR complexes. SUMM2 monitors perturbation of the MEKK1–MKK1/2–MPK4 signaling pathway [32] and, upon activation, promotes assembly of the downstream EDS1–PAD4–ADR1 signaling module [23]. Active SUMM2 further induces the higher-order clustering of ADR1-L1 resistosomes at the plasma membrane [23] (Figure 1B), consistent with the established Ca^2+^-permeable channel activity of activated helper RNLs [14] (Figure 2A). Thus, SUMM2 functions upstream of ADR1 resistosome assembly rather than acting as a canonical pore-forming CNL itself. These findings indicate that CNLs can promote immune signaling either by forming their own pore-forming resistosomes or by triggering the assembly of downstream helper-NLR resistosomes.

In contrast to pore-forming CNL resistosomes such as ZAR1, *Arabidopsis* TNLs activate immunity through a distinct mechanism centered on TIR-domain enzymatic signaling. Structural analysis of RPP1 revealed a tetrameric resistosome in which TIR domains form catalytic interfaces [13] (Figure 1B). Rather than directly forming membrane channels, TNLs generate immune-active nucleotide derivatives through TIR enzymatic activity [15,16]. Importantly, these products do not act equivalently on all EDS1-containing complexes. pRib-AMP and pRib-ADP preferentially bind the EDS1–PAD4 heterodimer and promote association with ADR1 family helpers, whereas ADPr-ATP and di-ADPR preferentially activate the EDS1–SAG101 module and its NRG1 partners [17,18]. Thus, EDS1–PAD4–ADR1 and EDS1–SAG101–NRG1 form biochemically distinct signaling branches rather than interchangeable downstream routes. Activated ADR1 and NRG1 proteins can subsequently assemble at the plasma membrane and form Ca^2+^-permeable channels [14,33] (Figure 2B), linking branch-selective decoding of TIR products to membrane-associated immune execution.

## 4. Networked Signaling Through Paired and Helper NLR Modules

Although some *Arabidopsis* NLRs integrate pathogen recognition and immune execution within a single receptor, many immune responses depend on coordinated interactions between multiple NLR components. Paired NLR systems represent an important organizational strategy in which recognition specificity and downstream signaling capacity are distributed between receptor partners. In these systems, one NLR primarily functions as a sensor that perceives pathogen effectors or effector-induced host alterations, whereas the partner receptor provides the signaling activity required for immune activation. The RRS1/RPS4 pair is a well-characterized example of this modular organization [19]. RRS1 contains an integrated WRKY domain that mimics host regulatory targets manipulated by pathogen effectors [10]. Effector interaction with or modification of the integrated WRKY domain is sensed by the RRS1–RPS4 receptor complex, relieving receptor repression and triggering RPS4-dependent immune signaling [34]. The CHS3/CSA1 system provides additional insight into how receptor compatibility influences NLR signaling. Genetic and biochemical analyses indicate that CHS3 and CSA1 alleles exhibit functional co-evolution and allele-specific compatibility [35]. CHS3–CSA1 pairs form resting-state heterodimers that undergo activation-dependent higher-order oligomerization (Figure 1B), with both conserved and allele-specific requirements for complex assembly [20]. Although the precise atomic organization of activated CHS3/CSA1 complexes remains unresolved, available evidence supports the idea that receptor association and higher-order assembly contribute to immune regulation.

Beyond paired receptor systems, *Arabidopsis* immunity relies on helper NLRs that provide shared signaling capacity for multiple upstream immune receptors. ADR1 and NRG1 represent two major helper-NLR families associated with distinct EDS1-dependent pathways [22]. The EDS1–PAD4 module promotes ADR1 activation, whereas the EDS1–SAG101 module activates NRG1-dependent signaling [29,36]. The modular organization of helper-NLR pathways also provides a point of convergence between cell-surface and intracellular immunity. The EDS1–PAD4–ADR1 node contributes directly to pattern-triggered immunity (PTI) [37], and efficient oligomerization of at least some helper NLRs requires coordinated activation of cell-surface and intracellular immune receptors [38]. These findings support more than simple downstream amplification: PRR-derived signaling can condition helper-NLR activation itself. However, a direct biochemical mechanism by which a defined PRR-derived signal controls resistosome assembly has not yet been established. PTI–ETI integration should therefore be viewed as a combination of mutual potentiation and shared signaling-node control rather than as two independent linear pathways. Representative *Arabidopsis* NLR assemblies, their molecular outputs, and functional principles are summarized in Table 1.

## 5. Higher-Order Organization of *Arabidopsis* Resistosome Assemblies

Structural information alone does not fully explain how NLR signaling is organized in living cells. Activated helper NLRs accumulate at the plasma membrane, and their localization, local concentration, and assembly state can influence channel activity and downstream signaling [14,33,38]. This spatial layer is especially important for distinguishing a structurally defined resistosome from larger signaling domains or clusters that emerge in vivo. Accordingly, higher-order organization should be treated as a regulatory property of NLR signaling rather than as evidence that every observed cluster represents a single stoichiometrically fixed supramolecular particle.

The SUMM2–ADR1-L1 pathway provides a useful example. SUMM2 monitors disruption of the MEKK1–MKK1/2–MPK4 signaling cascade [32,39] and promotes formation of an EDS1–PAD4–ADR1-L1 signaling module followed by higher-order ADR1-L1 clustering at the plasma membrane [23]. The clustering phenotype has been observed in planta and is linked genetically and functionally to SUMM2-dependent immune activation, supporting its physiological relevance. However, the available data do not establish that an ADR1-L1 cluster is a single particle with a fixed stoichiometry, nor do they yet define how cluster size quantitatively controls Ca^2+^ flux or cell-death thresholds. These distinctions are important when moving from structural snapshots to mechanisms operating in intact cells.

The next step is to connect assembly state with signaling dynamics. Key unresolved issues include when individual ADR1-L1 resistosomes nucleate into larger membrane domains, whether clustering changes channel open probability or persistence, how these structures are dismantled, and how spatial organization separates effective defense from damaging autoimmunity [23,33,40]. Addressing these questions will require time-resolved imaging in intact plants together with quantitative Ca^2+^ measurements, biochemical reconstitution, and genetic perturbation of cluster-forming interfaces.

## 6. Regulatory Principles Governing *Arabidopsis* NLR Activation Thresholds

Because activated NLR complexes can trigger strong immune outputs, including ion influx and programmed cell death, *Arabidopsis* must maintain precise control over receptor abundance, localization, and activation competence [12,14]. Regulation occurs at multiple levels, including transcription, protein stability and folding, subcellular trafficking, and post-translational modification [41,42,43,44]. Phosphorylation, ubiquitination, lipidation, acetylation, and SUMOylation can alter receptor stability, localization, conformational dynamics, and partner interactions [43]. These processes collectively set the threshold for productive immune activation: insufficient signaling compromises defense, whereas excessive or spontaneous activation can impose cell-death and growth penalties [25,44].

NLR activation thresholds are also embedded in broader stress, hormone, and transcription-factor networks. Transcript-level control is an established component of NLR regulation [44]. In *Arabidopsis*, β-aminobutyric acid (BABA)-induced resistance is accompanied by coordinated changes in salicylic acid, jasmonic acid, abscisic acid, and ethylene-associated transcriptional programs and by altered activity of candidate master regulatory transcription factors [45], illustrating how immune priming can reshape the signaling context in which NLR pathways operate. Recent promoter-motif profiling in *Arabidopsis* further demonstrates how transcription-factor binding-site distributions can be used to infer auto- and cross-regulatory relationships [46], an approach that could be extended directly to NLR promoters. These studies do not establish that every BABA-responsive transcription factor directly regulates an NLR gene; instead, they highlight a testable framework linking hormone state, promoter occupancy, NLR abundance, and subsequent resistosome competence.

Subcellular localization provides another critical regulatory layer. The requirement for N-myristoylation in SUMM2-mediated signaling shows that membrane targeting is necessary for organizing downstream immune complexes [23], while ADR1 and NRG1 require appropriate plasma-membrane association to assemble functional channels [14,33]. Regulation also occurs through signaling interference between NLR components. NRG1C provides a concrete example: this truncated helper NLR antagonizes full-length NRG1A/NRG1B and interferes with productive helper-NLR signaling complexes [47,48,49]. Paired-receptor compatibility and branch-selective EDS1 interactions impose additional checkpoints [17,18,29,50]. Thus, receptor abundance, localization, partner compatibility, and inhibitory competition jointly determine whether an activation signal progresses to full immune execution.

The final constraint on NLR activation is the balance between pathogen resistance and plant fitness [44,51,52,53]. Although strong immune responses can restrict pathogen proliferation, excessive or constitutive NLR activity often imposes substantial physiological costs. Therefore, evaluation of engineered or naturally occurring NLR variants requires consideration of long-term effects on plant growth and development rather than relying solely on short-term immune outputs [52,53,54,55]. Achieving durable resistance requires an appropriate balance among receptor abundance, activation threshold, signaling strength, and physiological cost [44,52,53]. Understanding these regulatory principles will be essential for interpreting natural NLR variation and designing immune receptors with predictable activity [26,54,55].

## 7. Engineering *Arabidopsis* NLR Recognition and Signaling Modules

The increasing structural and mechanistic understanding of NLRs has created several routes for receptor engineering, but the level of experimental validation differs substantially among them (Table 2). Structure-guided alteration of recognition surfaces and engineering of integrated domains have already expanded effector recognition or disease-resistance profiles in crop NLRs [56,57,58]. Nanobody–NLR fusions have also provided proof of concept that an artificial binding module can be coupled to an NLR scaffold to confer resistance against engineered plant viruses [59]. By contrast, direct tuning of resistosome stoichiometry, channel conductance, or helper–NLR coupling remains largely prospective. These approaches should therefore not be presented as equivalent stages of technology readiness.

For all engineering strategies, broadened recognition is only one criterion of success. Receptor folding, sensor–executor or sensor–helper compatibility, activation thresholds, and the risk of spontaneous autoimmunity must be evaluated together [26,52,55]. Structural or transient-expression evidence is not sufficient to establish agronomic utility: engineered receptors require in planta testing for pathogen restriction, durability, dependence on native partners, and growth or reproductive penalties over the plant life cycle. A practical design objective is therefore not maximal immune output, but to find a receptor that remains quiescent in the absence of pathogen challenge and activates strongly enough to confer resistance without a measurable fitness cost.

## 8. Outstanding Questions and Future Perspectives

Although structural studies have defined several activated NLR states [11,13,27,28], the principal gap is now to explain how these states are entered, maintained, and terminated in living plants. Static structures should therefore be paired with time-resolved in planta measurements of assembly kinetics, Ca^2+^ flux, transcriptional output, and cell fate. For TNL signaling, a priority is to determine how the abundance and localization of distinct TIR-derived molecules control competition or partitioning between the EDS1–PAD4–ADR1 and EDS1–SAG101–NRG1 branches. For helper NLRs, quantitative tests of membrane clustering and channel activity are needed to determine whether higher-order organization amplifies signal strength, prolongs signaling, or changes activation thresholds.

A second priority is to establish which principles learned in *Arabidopsis* can be transferred to crops. Some mechanisms appear broadly conserved: wheat resistosome structures support common principles of membrane-associated immune-receptor channels [60], and recognition engineering has been functionally demonstrated in rice NLRs [57,58]. At the same time, oligomeric architecture can differ across species; the octameric CC_G10_-NLR resistosome described in wheat [61] should not be assigned to *Arabidopsis* RPS2. Sensor–helper compatibility, EDS1-branch usage, and the fitness consequences of receptor activation may also be lineage- or genotype-specific. Translation to crop protection should therefore proceed receptor by receptor, combining structural design with in planta validation of partner dependence, disease resistance, and growth performance. This framework also argues for inducible or threshold-tuned engineering strategies that widen recognition without constitutive immune activation.

Together, these priorities shift the field from cataloguing receptor structures toward predictive control of immune behavior: defining which molecular features determine assembly state, which cellular signals set activation thresholds, and which engineered changes remain effective without compromising plant fitness.

**Outstanding questions**

1. Which sequence and structural features determine whether an activated NLR forms a defined resistosome, a transient intermediate, or a larger membrane-associated cluster?
2. How do the kinetics of NLR oligomerization, membrane recruitment, Ca^2+^ flux, and complex disassembly determine the magnitude and duration of immune output in planta?
3. How are pRib-AMP/ADP and ADPr-ATP/di-ADPR spatially and quantitatively partitioned between the EDS1-PAD4-ADR1 and EDS1-SAG101-NRG1 branches?
4. Which PRR-, hormone-, and transcription-factor-derived signals directly alter helper-NLR activation or resistosome competence, rather than only amplifying downstream defense outputs?
5. What is the causal contribution of higher-order ADR1-L1 clustering to channel activity, defense efficacy, and cell-death thresholds in intact plants?
6. Which NLR-engineering strategies retain enhanced disease resistance across crop genotypes and environments without autoimmunity, partner incompatibility, or measurable fitness penalties?

## 9. Conclusions

Three conclusions emerge from current work on *Arabidopsis* NLR immunity. First, higher-order assembly is a recurring activation principle, but the biochemical consequences of assembly are class- and receptor-specific: pore formation is established for selected CNL and RNL systems, whereas TNLs function as catalytic signal generators that rely on branch-selective EDS1–helper modules. Second, immune output is determined not only by receptor structure but also by partner compatibility, membrane localization, signaling interference, transcriptional state, and spatial organization in living cells. Third, NLR engineering is already experimentally feasible at the recognition level, including integrated-domain redesign and nanobody-based recognition, but durable plant protection will depend on coupling broadened recognition to controlled activation. The most useful next-generation receptors are therefore likely to combine validated recognition modules with native-compatible signaling partners and activation thresholds that minimize constitutive defense costs. Progress toward this goal will require structural design to be tested alongside quantitative in planta measurements of signaling dynamics, pathogen restriction, and whole-plant fitness, followed by validation in crop backgrounds rather than assuming direct transfer from *Arabidopsis*.

## Figures and Tables

**Table 1:** Representative *Arabidopsis* NLR assemblies and molecular outputs.

NLR System	NLR Class/Role	Activated Assembly	Molecular Output	Functional Principle
ZAR1	CNL sensor/executor	Pentameric resistosome (resolved)	Ca^2+^-permeable cation channel	Direct membrane channel formation
RPP1	TNL sensor	Tetrameric resistosome (resolved)	TIR-domain enzymatic signaling	Generation of branch-selective TIR signaling molecules
ADR1	RNL helper	Activated helper-NLR resistosome	Ca^2+^ influx	Execution of EDS1–PAD4-dependent signaling
NRG1	RNL helper	Activated helper-NLR resistosome	Ca^2+^ influx	Execution of EDS1–SAG101-dependent signaling
CHS3/CSA1	Paired TNL receptors	Activation-dependent higher-order assembly (atomic architecture unresolved)	TIR-dependent signaling	Receptor compatibility and immune regulation
SUMM2–ADR1-L1	CNL sensor + RNL helper module	Higher-order ADR1-L1 clusters (cluster stoichiometry unresolved)	Membrane-associated immune activation	Spatial organization of helper-NLR signaling

A comparison across these systems separates a conserved activation logic from class-specific execution. Release of autoinhibition, nucleotide-dependent conformational change, and higher-order assembly recur across NLR classes, but their outputs are not equivalent. ZAR1 and selected RNL helpers execute immunity through membrane-associated cation-channel activity, whereas TNL resistosomes function primarily as catalytic signal generators and depend on EDS1–helper modules for downstream execution. Oligomerization can therefore be regarded as a broadly recurring principle, while pore formation is presently demonstrated only for particular CNL and RNL systems and should not be generalized to every receptor in those classes.

**Table 2:** Validation status, representative strategies, and major constraints for engineering NLR immune receptors.

Strategy	Concept	Representative Approach/Evidence Status	Major Consideration
Structure-guided modification	Alter recognition interfaces while maintaining receptor architecture	Effector-recognition surface redesign; experimentally validated in crop NLRs [57]	Preserve conformational control and activation threshold
Integrated-domain engineering	Utilize modular recognition domains within NLR scaffolds	Integrated-domain redesign; experimentally validated in rice [56,58]	Maintain receptor folding and signaling compatibility
Synthetic recognition fusion	Introduce artificial binding modules into NLR frameworks	Nanobody–NLR fusions; proof-of-concept disease resistance [59]	Ensure integration with downstream signaling machinery
Programmable immune receptor design	Combine designed recognition modules with NLR signaling systems	Designed recognition–signaling combinations; emerging/prospective	Balance recognition specificity, activation control, and physiological fitness

## Data Availability

No new data were created or analyzed in this study. Data sharing is not applicable to this article.
